# The action of high K^+^ and aglycaemia on the electrical properties and synaptic transmission in rat intracardiac ganglion neurones *in vitro*

**DOI:** 10.1113/expphysiol.2008.044784

**Published:** 2008-10-31

**Authors:** Jhansi Dyavanapalli, Katrina Rimmer, Alexander A Harper

**Affiliations:** Division of Molecular Physiology, College of Life Sciences, University of DundeeDundee DD1 5EH, UK

## Abstract

We have investigated the action of two elements of acute ischaemia, high potassium and aglycaemia, on the electrophysiological properties and ganglionic transmission of adult rat intracardiac ganglion (ICG) neurones. We used a whole-mount ganglion preparation of the right atrial ganglion plexus and sharp microelectrode recording techniques. Increasing extracellular K^+^ from its normal value of 4.7 mm to 10 mm decreased membrane potential and action potential after-hyperpolarization amplitude but otherwise had no effect on postganglionic membrane properties. It did, however, reduce the ability of synaptically evoked action potentials to follow high-frequency (100 Hz) repetitive stimulation. A further increase in K^+^ changed both the passive and the active membrane properties of the postganglionic neurone: time constant, membrane resistance and action potential overshoot were all decreased in high K^+^ (20 mm). The ICG neurones display a predominantly phasic discharge in response to prolonged depolarizing current pulses. High K^+^ had no impact on this behaviour but reduced the time-dependent rectification response to hyperpolarizing currents. At 20 mm, K^+^ practically blocked ganglionic transmission in most neurones at all frequencies tested. Aglycaemia, nominally glucose-free physiological saline solution (PSS), increased the time constant and membrane resistance of ICG neurones but otherwise had no action on their passive or active properties or ganglionic transmission. However, the combination of aglycaemia and 20 mm K^+^ displayed an improvement in passive properties and ganglionic transmission when compared with 20 mm K^+^ PSS. These data indicate that the presynaptic terminal is the primary target of high extracellular potassium and that aglycaemia may have protective actions against this challenge.

Neural control of the heart is under the influence of the sympathetic and the parasympathetic divisions of the autonomic nervous system. Activation of the parasympathetic division, arising from neurones in the medulla of the brainstem, has negative chronotropic, dromotropic and inotropic actions (Adams & Cuevas, 2004). The intracardiac ganglia (ICG) form the final common pathway for the cardiac autonomic nervous system. While traditionally the intrinsic cardiac nervous system was held as a simple relay for parasympathetic inputs to the cardiac end-effectors, recent work has clearly indicated that these peripheral ganglia are complex neural networks, with multiple neuronal subtypes, capable of complex reflex control of regional cardiac function, even when disconnected from the central nervous system (Ardell, 2004; Armour, 2008).

Blockage of a coronary artery will result in an inadequate blood flow (ischaemia) downstream from the occlusion. Ischaemia generates a multifaceted challenge (Carmeliet, 1999). Acute myocardial ischaemia leads to derangements in cellular electrical stability and the generation of lethal arrhythmias. Several factors underpin these changes. Hydrolysis of high-energy phosphate compounds (ATP and phosphocreatine) releases large amounts of phosphate in the ischaemic heart. This, together with K^+^ and lactate produced by anaerobic glycolysis, passes into the extracellular space (Katz, 2006). Extracellular [K^+^] in the affected region can quickly accumulate to values approaching 20 mm (Carmeliet, 1999).

The proximity of the ICG to the coronary blood supply makes it susceptible to the effects of ischaemia and reperfusion associated with myocardial infarct (Horackova & Armour, 1995; Armour, 1999). Each ganglionated plexus in the canine heart is perfused by two or more arterial branches that arise from different major coronary arteries (Huang *et al.* 1993).

While the effects of ischaemia and reperfusion on cardiac cell function have been the subject of many studies (Toda, 1969; Kourie, 1998; Carmeliet, 1999; Rodriguez *et al.* 2006), there are few reports of their action on the intrinsic cardiac nervous system. The negative chronotropic action of vagal activation was blunted in a rat model of myocardial infarction (Du *et al.* 1998), and the release of acetylcholine during vagal stimulation was abrogated by ischaemia in the anaesthetized dog (Kawada *et al.* 2002). Accordingly, conditions simulating ischaemia, for example high K^+^, also attenuated the negative chronotropic action of vagal activation in the rat (Sears *et al.* 1999). High K^+^ reduced neuronal activity in canine ICG neurones *in situ*, measured using focal extracellular recording techniques (Thompson *et al.* 2000*b*).

The principal targets for the action of ischaemia within the ICG are: (i) synaptic transmission, namely, preganglionic terminal and postganglionic receptors; and (ii) the encoding properties of the postganglionic neurone.

Here we report the results of experiments exploring the action of two elements of ischaemia, high extracellular potassium and aglycaemia, applied individually and together, on the electrophysiological properties and ganglionic transmission in rat ICG neurones.

## Methods

### Preparation

Development of a whole-mount ganglion preparation from adult rats has been described previously (Rimmer & Harper, 2006). Briefly, young female adult Wistar rats (≥6 weeks, 100–170 g) obtained from Harlan, Bicester, UK were killed by stunning and cervical dislocation, in accordance with current UK Home Office guidelines. The heart and lungs were quickly excised and the atria were isolated. Most of the underlying atrial muscle was removed to leave a section of atria, ∼5 mm × 5 mm, comprising the epicardium containing the right atrial ganglion plexus and vagosympathetic trunk. The preparation did not include the sinoatrial node because spontaneous beating would dislodge the recording microelectrode.

The neurochemical profile of this preparation has been analysed, all principal neurones being immunoreactive to choline acetyl-transferase (Richardson *et al.* 2003), indicating that they release acetylcholine. Recordings were normally made from the large ganglion located at the junction of the right superior vena cava and right atrium. Preferential control of specific cardiac functions has been attributed to specific ganglia. In the rat, the right atrial ganglion plexus neurones are primarily associated with regulation of sinoatrial node function (Sampaio *et al.* 2003).

The whole-mount preparation comprising ganglion and epicardium was pinned out in a recording chamber (∼1.0 ml volume) and superfused with bicarbonate-buffered physiological saline solution (PSS) at 2 ml min^−1^ (Gilson Minipuls 3; Digitimer, Herts, UK). The temperature of the superfusing solution was controlled by a Peltier heating device (Medical systems PDMI-2 micro incubator; YSI 400, Yellow Springs, OH, USA) to 35°C, monitored by an independent thermistor probe in the recording chamber. The tissue was left to recover in these conditions for ∼30 min before commencing recording. The ICG neurones were visualized using differential interference contrast optics on a fixed stage microscope.

### Electrophysiological recordings

Intracellular recordings from postganglionic ICG somata were made using sharp microelectrodes pulled from borosilicate glass (GC120F; Harvard Apparatus Ltd, Edenbridge, UK) with resistances of ∼120 MΩ when filled with 0.5 m KCl. Membrane voltage responses were recorded with a conventional bridge amplifier (Axoclamp 2A, ×0.1 LU headstage, Axon Instruments Inc., Union City, CA, USA). Voltage signals were filtered at 20 kHz (Frequency Devices 902, Cambridge, UK), digitized at 50 kHz and transferred to a Pentium 4 computer using an analog-to-digital converter (Micro 1401 MKII interface, Cambridge Electronic Design, Cambridge, UK) and Spike2 version 6 software (Cambridge Electronic Design).

Before penetrating the neurone, a +0.2 nA pulse (20 ms duration, 5 Hz) was injected to optimize the rise time of the voltage trace (without overshoot), normally ≤0.1 ms using capacity compensation. Bridge balance was then applied to remove the instantaneous voltage step due to the voltage drop across the microelectrode resistance. It is a matter of concern in single microelectrode studies how closely the current pulse injected corresponds to that intended, particularly with high-resistance electrodes. The intracellular electrodes used in these experiments normally exhibited no manifest change in microelectrode resistance during current injection, as indicated by good balancing characteristics (≤±0.3 nA). Following impalement of the neurone, bridge balance was adjusted, if required, using the method described by Engel *et al.* (1972), to achieve a smooth change in membrane potential at the onset of the current pulse.

Two types of current clamp protocol were routinely performed. In the first, brief intracellular depolarizing currents (≤3 ms in duration) were used to directly evoke single somatic action potentials. Action potential parameters measured were overshoot, after-hyperpolarization (AHP) amplitude and duration to 50% recovery (AHP_50_), using a Spike2 script. Long (500 ms) hyperpolarizing and depolarizing pulses were applied to measure input resistance (*R*_in_), time constant (τ) and time-dependent rectification, and evoked discharge characteristics, respectively. Input resistance (*R*_in_) was calculated from the steady state of the voltage response to small hyperpolarizing, long current pulses (≤−0.1 nA). Time constant was measured at small hyperpolarizing current pulses (≤−0.1 nA) using Spike2 software. Membrane resistance (*R*_m_) was calculated from the time constant (τ=*R*_m_×*C*_m_, where *C*_m_ is the specific membrane capacitance, assumed to be 1 μF cm^−2^). Current pulses (+0.02 to +0.3 nA) were applied to determine threshold current. Discharge activity was classified as being phasic, multiple adapting or tonic upon application of a long depolarizing current pulse of roughly twice threshold intensity (Rimmer & Harper, 2006).

Branches of the vagus and interganglionic nerve trunks were stimulated using a glass suction electrode connected to a constant voltage isolated stimulator (Digitimer DS2, Digitimer, Herts, UK). Nerve trunks were stimulated using stimulus pulses of 0.02–0.2 ms width, 5–50 V amplitude. Acetylcholine was focally applied using a pressure-ejection device (200 kPa; Picospritzer II, General Valve, Fairfield, NJ, USA), with the pressure ejection pipette positioned ≤50 μm from the neuronal soma to maximize the response to agonist application.

### Solutions and pharmacological agents

The PSS contained (mm): 118 NaCl, 25 NaHCO_3_, 1.13 NaH_2_PO_4_, 4.7 KCl, 1.8 CaCl_2_, 1.3 MgCl_2_ and 11.1 glucose and was gassed (continuously) with 95% O_2_–5% CO_2_ to pH 7.4 (Smith *et al.* 2001). Alterations in extracellular K^+^ concentrations were made by equimolar substitution of K^+^ for Na^+^. Nominally zero-glucose conditions were achieved by replacing glucose in PSS with isosmolar sorbitol (Birinyi-Strachan *et al.* 2005).

Different PSS solutions (high K^+^, nominally zero glucose (aglycaemia) and high K^+^ with nominally zero glucose) were applied for 20 min before making recordings. This length of time has been used to simulate ischaemia in the isolated perfused working rat heart (Khogali *et al.* 1998). All reagents were of analytical grade.

The concentration of glucose in the superfusing PSS was determined using a hexokinase assay kit (Sigma).

### Electrophysiological equations

With the caveat that there is no significant direct contribution from the Na^+^ pump, the resting membrane potential (*E*_m_) is given by the Goldman–Hodgkin–Katz (GHK) equation, as follows:

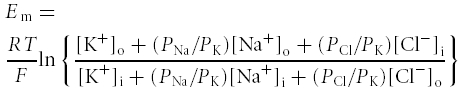
where *R* and *F* have their usual meanings and values, *T*= 308K, and *P* is the permeability of the specified ion.

In IGC neurones, the Na^+^ pump makes only a minor electrogenic contribution to the resting membrane potential (Xu & Adams, 1992; J. Dayavanapalli, K. Rimmer and A. A. Harper, unpublished observations). The term (*P*_Na_/*P*_K_)[Na^+^]_i_ can also be taken to be negligible. Additionally, the resting Cl^−^ conductance is very small (Xu & Adams, 1992; Fischer *et al.* 2005). Therefore, the (*P*_Cl_/*P*_K_)[Cl^–^]_o_ and (*P*_Cl_/*P*_K_)[Cl^–^]_i_ terms can also be regarded as making no significant input.

This equation can then be rearranged into the linear form:

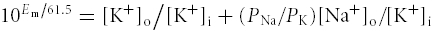


This can be plotted as a straight line with a slope of 1/[K^+^]_i_ and an intercept on the ordinate of (*P*_Na_/*P*_K_)[Na^+^]_o_/[K^+^]_i_. Thus, from measurements of *E*_m_ at different values of [K^+^]_o_, the two unknowns in eqn (1), *P*_Na_/*P*_K_ and [K^+^]_i_, can be calculated (Brown *et al.* 1999).

Data are presented as the means ±s.d., and were compared using ANOVA (Tukey–Kramer multiple comparisons test) and Student's paired *t* tests (SigmaStat 3.1, Systat Software Inc., London, UK).

## Results

### General properties of ICG neurones

All results presented are from ICG neurones with a resting membrane potential ≥−40 mV and overshooting somatic action potentials elicited by short depolarizing current pulses (2–3 ms) in control conditions. Recordings were stable for at least 10 min before readings were taken and the superfusing PSS solution was altered.

The mean resting membrane potential was −49.7 mV (±6.7 mV, *n*= 28). The input resistance of ICG neurones ranged from 49 to 235 MΩ, with a mean value of 116 MΩ (±46 MΩ, *n*= 28) and the mean time constant was 6.5 ms (±3.0 ms, *n*= 28). These values are in good agreement with those reported for adult rat ICG neurones in intact ganglia in comparable experimental conditions (Selyanko, 1992; Rimmer & Harper, 2006). The neurone capacitance ranged from 20 to 108 pF, with a mean value of 60 pF (±25 pF, *n*= 28). Autonomic ganglion neurones can be differentiated on the basis of the discharge characteristics of the soma membrane to depolarizing current pulses (Adams & Harper, 1995; Adams & Cuevas, 2004). The evoked discharge activity of the neurones in this study was predominantly phasic (16/28 neurones), the remainder being classified as either multiple adapting (11 neurones) or tonic (1 neurone).

Synaptic transmission was normally secure over the normal frequency range recorded during efferent reflex discharge ≤50 Hz (Kunze, 1972).

### The action of altering potassium on the membrane properties of the postganglionic neurone

#### Passive properties

The effect of reducing and increasing [K^+^]_o_ on *E*_m_ was explored to better characterize its action. As external K^+^ was decreased from its control value of 4.7 mm to 1 mm, the resting membrane potential hyperpolarized slightly; increasing K^+^ stepwise from 4.7 up to 50 mm depolarized the membrane potential.

**Figure 1:**
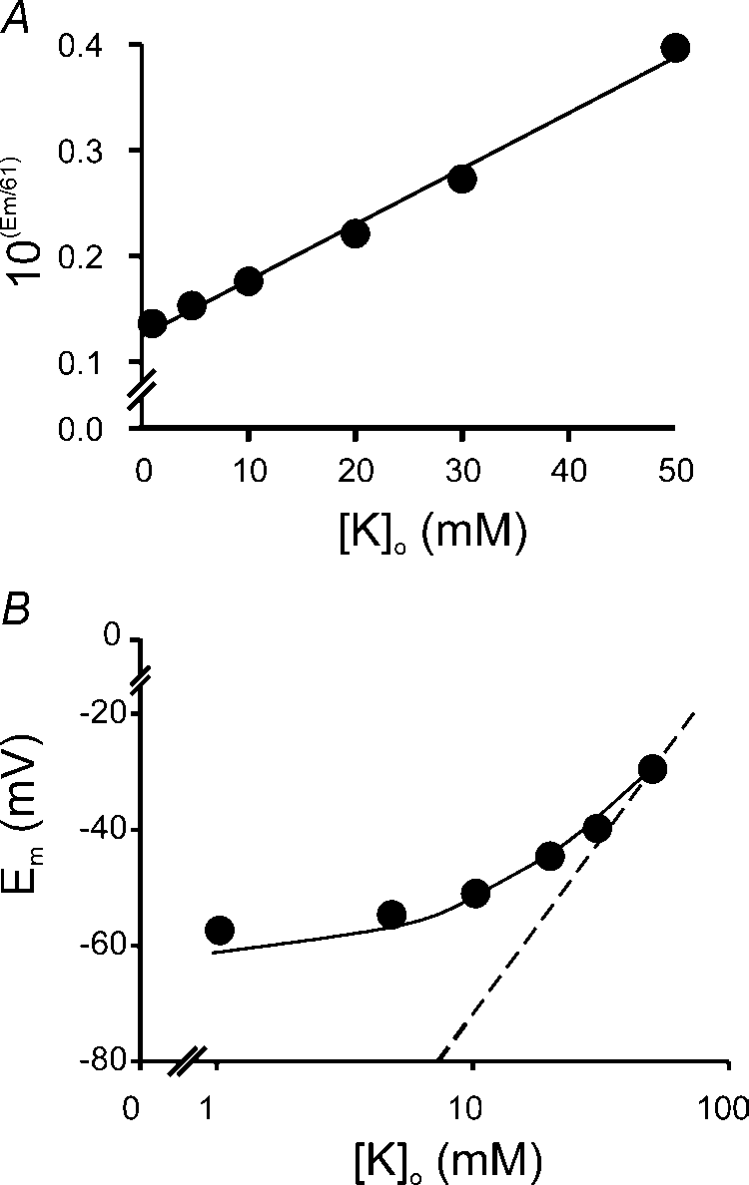
The effect of reducing and increasing [K^+^]_o_ on resting membrane potential *A*, plot of 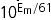 against [K^+^]_o_. In this neurone, *P*_Na_/*P*_K_ was calculated to be 0.12 and [K^+^]_i_ 190 mm and the linear regression coefficient (*r*^2^) was 0.99; see Methods, eqn (2). Representative of 6 experiments. *B*, resting values of *E*_m_ are plotted as a function of [K^+^]_o_ (logarithmic scale) in normal PSS. The data are fitted with the GHK equation, eqn (1), with a *P*_Na_/*P*_K_ of 0.13. The straight line was drawn according to the Nernst equation for a K^+^-selective electrode, slope of 61 mV per decade change in [K^+^]_o_.

The relationship between 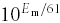 and [K^+^]_o_ was plotted, and results of a typical experiment are presented in Fig. 1*A*. The *E*_m_ data are also presented as a function of [K^+^]_o_ and fitted with the GHK equation in Fig. 1*B*. In all six such experiments analysed using this scheme, there was a good linear correlation between 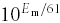 and [K^+^]_o_ (correlation coefficient, *r*^2^≥ 0.95). The *P*_Na_/*P*_K_ ratio was 0.13 (±0.02, *n*= 6); this value compares satisfactorily with published estimates of the relative permeability to Na^+^ (*P*_Na_/*P*_K_) obtained using the GHK equation (Cuevas *et al.* 1997; Hogg *et al.* 2001). The mean [K^+^]_i_ was 197 mm (±15 mm, *n*= 6) agreeing with the sparse data available on [K^+^]_i_ in autonomic ganglia (predominantly for superior cervical sympathetic ganglion neurones, reviewed by Adams & Harper, 1995).

There were limited data points at high [K^+^]_o_ (>20 mm) because of dislodgement of the recording microelectrode owing to depolarization-induced contraction of the underlying atrial musculature.

The mean *R*_in_ was reduced, but not significantly, in high-[K^+^]_o_ solutions. The time constant, hence membrane resistance, were, however, significantly decreased (see Table 1). This apparent discrepancy is almost certainly due to inadequate compensation for increases in microelectrode tip resistance during some experiments, hence overestimation of *R*_in_. This artefact does not impact on the calculation of *R*_m_.

**Table 1:** Actions of high K^+^ on the passive and active electrical properties of adult ICG neurones

	4.7 mm[K^+^]_o_	10 mm[K^+^]_o_	20 mm[K^+^]_o_
Passive properties			
*E*_m_ (mV)	−48.0 ± 4.9 (6)	−41.5 ± 6.7 (6)†	−36.9 ± 8.9 (6)**‡
*R*_in_ (MΩ)	85.4 ± 27 (6)	82.3 ± 25 (6)^n.s.^	71.7 ± 13.4 (6)^n.s.^
*R*_m_ (kΩ cm^2^)	5.4 ± 2.0 (6)	4.6 ± 1.6 (6)	2.0 ± 1.0 (6)**‡
τ (ms)	5.4 ± 2.0 (6)	4.6 ± 1.6 (6)	2.0 ± 1.0 (6)**‡
Active properties			
AP overshoot (mV)	20 ± 5.0 (6)	16.0 ± 7.6 (6)	12.0 ± 6.2 (6)*
AHP amplitude (mV)	15.4 ± 3.5 (6)	9.5 ± 1.3 (6)††	4.8 ± 1.8 (6)***‡‡
AHP_50_ (ms)	16.8 ± 5.4 (6)	18.2 ± 6.8 (6)^n.s.^	25.8 ± 21 (6)^n.s.^

Values are means ±s.d.; number of neurones in parentheses. Repeated Measures-ANOVA was used to test the significance between the groups.

* *P* < 0.05,

** *P* < 0.01 and

*** *P* < 0.005 for 4.7 mm*versus* 20 mm[K^+^]_o_;

† *P* < 0.05 and

†† *P* < 0.01 for 4.7 mm*versus* 10 mm[K^+^]_o_;

‡ *P* < 0.05 and

‡‡ *P* < 0.01 for 10 mm*versus* 20 mm[K^+^]_o_; and n.s., not significant.

**Figure 2:**
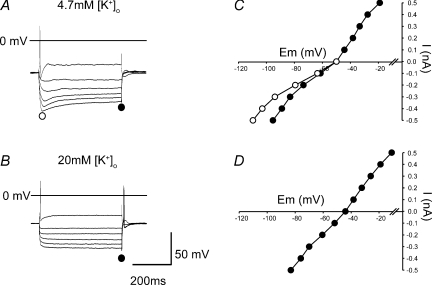
Membrane potential response to depolarizing and hyperpolarizing current pulses *A* and *B*, voltage responses obtained in response to depolarizing and hyperpolarizing current pulses (+0.2 nA and −0.1 to −0.5 nA) are shown for control conditions and in high [K^+^]_o_ (20 mm). *C* and *D*, current–voltage (*I–V*) relations for the conditions in *A* and *B*, respectively, plotted for the peak voltage response (⊚) and the steady-state response measured at the end of the current step (•).

Hyperpolarizing current pulses can induce time-dependent rectification (TDR), held as the signature of the H-current (Pape, 1996). Such behaviour was observed to varying extents in all neurones but was either blunted or absent in high-[K^+^] PSS; see Fig. 2. The amount of TDR was quantified by measuring the steady-state voltage response to a hyperpolarizing current pulse to approximately −90 ± 10 mV and expressing this as a percentage of peak, with time, membrane potential excursion. The values were 92.0 ± 3.9 and 101 ± 1.5% (*n*= 8) for ICG neurones in 4.7 and 20 mm[K^+^], respectively. The values in high [K^+^]_o_ were significantly different from those of control neurones (*P*= 0.001, Student's paired *t* test).

#### Active properties

**Figure 3:**
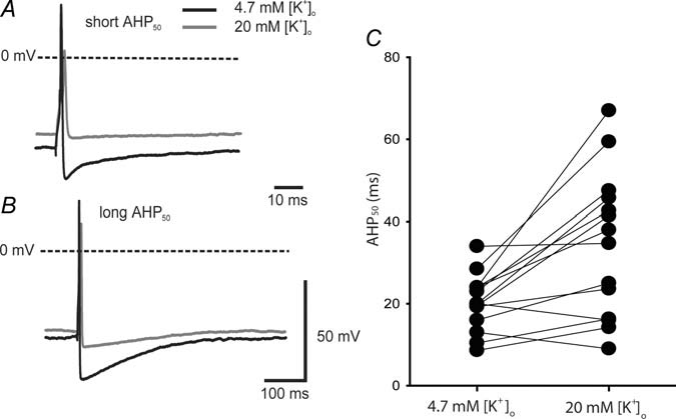
The action of high [K^+^]_o_ (20 mm) on somatic action potential characteristics Somatic action potential traces produced by brief depolarizing current pulses from ICG neurones with short (*A*) and long AHP_50_ durations (*B*) in control and 20 mm K^+^ PSS. Top time calibration bar refers to *A* and bottom bar refers to *B*. *C*, AHP_50_ values in 4.7 and in 20 mm K^+^; data taken from Tables 1 and 3 (*n*= 14).

Action potential parameters measured were its overshoot and the after-hyperpolarization (AHP) following the action potential, characterized by its amplitude and decay time. Adult ICG neurones had action potentials (APs) with large AHP amplitudes (17 ± 6 mV, *n*= 22) and a wide range of durations (9–62 ms), in good agreement with previous reports (Rimmer & Harper, 2006). The action of 20 mm K^+^ was dependent on AHP duration as gauged by time to 50% recovery (AHP_50_; Edwards *et al.* 1995); see Tables 1 and 3. The AHP_50_ values recorded in 4.7 and 20 mm K^+^ are plotted in Fig. 3*C*. High K^+^ had no apparent action on short AHP_50_ values but progressively increased the duration of longer AHP_50_ values. The overshoot and AHP amplitude decreased with increasing [K^+^]_o_; see Table 1 and Fig. 3. The increase in [K^+^]_o_ was compensated for by a decrease in [Na^+^]_o_. The decrease in overshoot amplitude was, however, greater than can be accounted for by the expected shift in *E*_Na_ (∼−3 mV for 20 mm K^+^ PSS), presumably reflecting increased Na^+^ channel inactivation accompanying membrane potential depolarization in this solution. The absolute amplitude of the AHP changed by ∼20 mV (from −63 to −45 mV in 4.7 and 20 mm K^+^ PSS, respectively) tracking the calculated decrease in *E*_K_ (∼38 mV). This action was similar in neurones displaying either long or short AHP_50_ values; see Fig. 3. Apart from a rare brief burst of action potentials in response to switching to high-K^+^ PSS, there was no evidence of any progressive change in AP parameters over the exposure time. The evoked responses to long depolarizing pulses were unaffected by high-K^+^ solutions, remaining predominantly phasic; see Fig. 2.

**Table 3:** Actions of high K^+^ with or without **aglycaemia on the passive and active membrane properties of adult ICG neurones**

	4.7 mm[K^+^]_o_	20 mm[K^+^]_o_	20 mm[K^+^]_o_, aglycaemia
Passive properties			
*E*_m_ (mV)	−49.6 ± 4.5 (11)	−42.5 ± 3.5 (11)***	−45.8 ± 4.9 (11)††
*R*_m_ (kΩ cm^2^)	7.8 ± 3.3 (11)	2.9 ± 1.0 (11)***	3.3 ± 1.3 (11)†
τ (ms)	7.8 ± 3.3 (11)	2.9 ± 1.0 (11)***	3.3 ± 1.3 (11)†
Active properties			
AP overshoot (mV)	20.0 ± 5.4 (8)	13.3 ± 6.0 (8)*	13.2 ± 5.4 (8)
AHP amplitude (mV)	20.7 ± 4.6 (8)	8.1 ± 5.0 (8)***	7.5 ± 4.8 (8)†
AHP_50_ (ms)	23.0 ± 6.4 (8)	40.7 ± 11.5 (8)**	36 ± 18.1 (8)

Values are means ±s.d.; number of neurones in parentheses. Repeated measures-ANOVA was used to test the significance between the groups.

* *P* < 0.05,

** *P* < 0.01 and

*** *P* < 0.005 for 4.7 mm*versus* 20 mm[K^+^]_o_;

† *P* < 0.05 and

†† *P* < 0.01 for 20 mm[K^+^]_o_*versus* 20 mm[K^+^]_o_ with aglycaemia; and n.s., not significant.

### Ganglionic transmission

To be included in this component of the study, ICG neurones had to receive a strong synaptic input (indicated by supramaximal stimulation of the preganglionic nerve trunk evoking a suprathreshold EPSP and action potential in response to every stimulus at 0.2 Hz).

**Figure 4:**
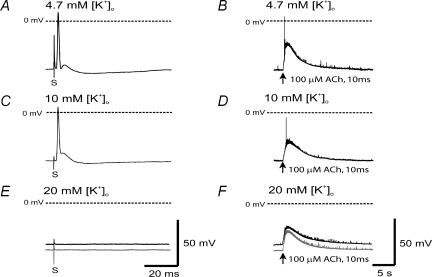
The action of increasing [K^+^]_o_ on single synaptically evoked APs (*A*, *C* and *E*) and excitatory ACh-evoked responses recorded from the same neurone (*B*, *D* and *F*) The effect of increasing [K^+^]_o_ from control 4.7 mm (*A*) to 10 mm (*C*) and 20 mm (*E*) on indirect nerve-evoked responses (stimulation applied at time ‘S’). Both 10 and 20 mm K^+^ depolarized *E*_m_ and in this instance 20 mm K^+^ blocked the synaptic response. The *E*_m_ in *E* was reset (grey trace) to native values recorded in normal PSS. *B*, focal application of ACh evoked transient depolarizing responses and action potential discharge. The ACh-evoked depolarization was unaffected by high [K^+^]_o_ values of 10 (*D*) and 20 mm K^+^ (*F*). In *F*, the *E*_m_ was reset (grey trace) to native values recorded in normal PSS.

**Figure 6:**
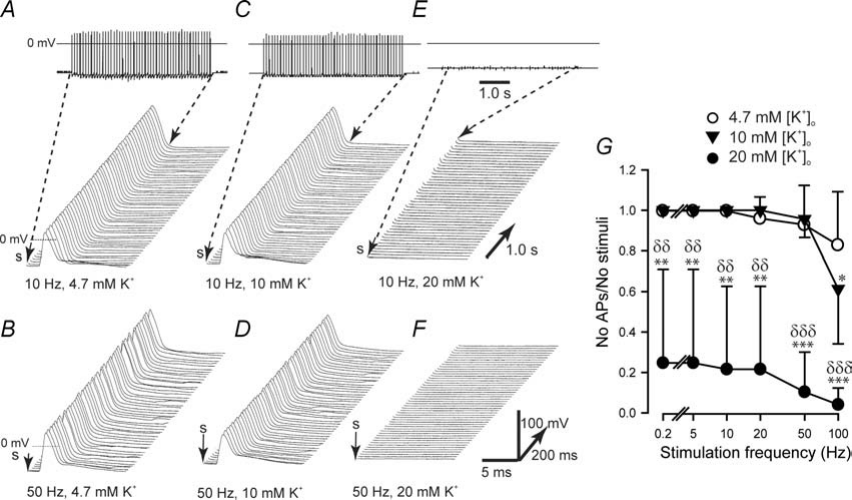
‘Waterfall’ display of action of high [K^+^]_o_ on synaptic transmission The effects of high K^+^ on synaptic transmission were investigated by applying trains of 50 stimuli applied at time ‘S’ (10 Hz for *A*, *C* and *E* and 50 Hz for *B*, *D* and *F*) to the preganglionic nerve trunk. The upper panels in *A*, *C* and *E* show voltage with time, records of individual volleys of pulses and the timing of the start and the end of the waterfall. *G*, the ratio of the number of successful somatic action potentials to the number of stimuli gives an index of the frequency dependence of ganglionic transmission (*P* < 0.01 at 0.2–20 Hz, *P* < 0.001 at 50–100 Hz, 4.7 *versus* 20 mm K^+^; *P* < 0.01 at 0.2–20 Hz, *P* < 0.001 at 50–100 Hz, 10 *versus* 20 mm K^+^, *n*= 8, RM-ANOVA). *P < 0.05, **P < 0.01 and ***P < 0.005 for 4.7 mm K^+^*versus* 20 mm K^+^; and δ*P* < 0.01 and δδ*P* < 0.005 for 10 mm K^+^*versus* 20 mm K^+^.

Application of 10 mm K^+^ PSS had little effect on ganglionic transmission evoked by either single or multiple trains of stimuli (see Figs 4 and 6).

The response to single stimuli was relatively insensitive to 10 mm K^+^, but was blocked completely in seven of 11 neurones following superfusion of 20 mm K^+^ PSS; Fig. 4. The post action potential depolarization in 10 mm K^+^ was larger than recorded in 4.7 mm K^+^. This was a consistent observation in five of five ICG neurones displaying strong synaptic transmission.

**Figure 5:**
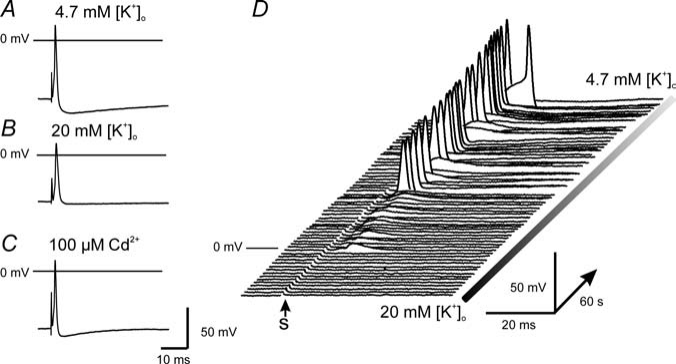
High [K^+^]_o_ (20 mm) does not influence antidromic conduction in the postganglionic axon and progressively block ganglionic transmission in ICG neurones *A* and *B*, nerve-evoked action potentials in control 4.7 mm K^+^ and high-[K^+^]_o_ (20 mm) PSS, respectively. *C*, Cd^2+^ (100 μm) does not block the nerve-evoked action potential (in 4.7 mm[K^+^]_o_), demonstrating that it is antidromic rather than synaptically mediated. *D*, there is a progressive recovery from synaptic block upon reducing the [K^+^]_o_ from 20 mm to the control value (4.7 mm); waterfall display of single stimuli (S) applied at 0.2 Hz while changing the [K^+^]_o_ of the superfusing PSS solution, indicated by the shaded bar.

The absence of postsynaptic responses in high-K^+^ PSS was not due to a block of axonal conduction or action of acetylcholine (ACh), as demonstrated by 20 mm K^+^ having no effect on antidromic conduction or focal application of ACh (100 μm, 5 ms), respectively; see Figs 4 and 5. When synaptic transmission failed upon increasing [K^+^]_o_, it was normally in an all-or-none fashion. However, upon reverting to 4.7 mm K^+^, recovery from block was progressive, so that subthreshold EPSPs were recorded before full action potentials, providing evidence that the block is not due to failure of conduction in the preganglionic axon; see Fig. 5*D*.

The ability of the postganglionic neurone to follow the activity of preganglionic stimuli at different frequencies was studied. Preganglionic parasympathetic axons discharge at 0–20 Hz at rest and 2–60 Hz with maximal activation (Kreulen, 2005). Trains of electrical stimuli, 20 or 50 pulses, of twice threshold voltage at frequencies up to 100 Hz were applied and action potential discharge monitored in the postganglionic neuronal soma. The ratio of number of successful action potentials to the number of stimuli was used to provide an index of the frequency dependence of ganglionic transmission. The results from a typical experiment are presented in Fig. 6. Ganglionic transmission was unaffected by 10 mm K^+^ over physiologically relevant frequencies (0.2–50 Hz). It was, however, significantly reduced at 100 Hz (*P*= 0.05, *n*= 8, Repeated measures-ANOVA); see Fig. 6. Further increasing [K^+^]_o_ to 20 mm significantly reduced ganglionic transmission over the entire frequency range studied. At the end of the manoeuvre, Cd^2+^ (100 μm) was always applied to test and substantiate that the response was synaptically mediated and not antidromic in nature; see Fig. 5.

### The action of aglycaemia

**Figure 7:**
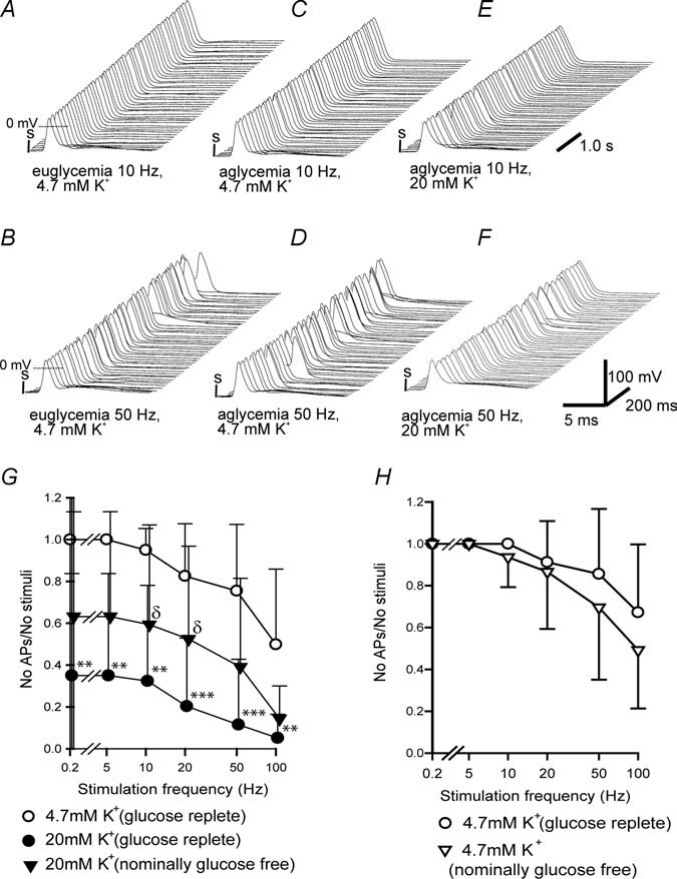
Aglycaemia partially abrogates the ganglionic transmission blocking action of high K^+^ The effects of aglycaemia and combined high K^+^ and aglycaemia on ganglionic transmission in the ICG. Trains of 50 stimuli applied at time ‘S’ (10 Hz for *A*, *C* and *E* and 50 Hz for *B*, *D* and *F*) were applied to the preganglionic nerve trunk whilst superfusing normal (*A* and *B*), nominally zero-glucose (aglycaemia; *C* and *D*) and nominally zero-glucose and 20 mm K^+^ PSS (*E* and *F*). *G*, the ratio of the number of successful somatic action potentials to the number of stimuli gives an index of the frequency dependence of ganglionic transmission in 4.7 mm K^+^, 20 mm K^+^ and 20 mm K^+^ with aglycaemic conditions (*n*= 11). *H*, frequency dependence of synaptic transmission in 4.7 mm K^+^ and nominally glucose-free conditions (*n*= 5). There was no difference in the frequency dependence of ganglionic transmission between glucose-replete and glucose-free PSS. In *G*, the symbols and error bars have been displaced horizontally to aid vizualization. **P < 0.01 and ***P < 0.005 for 4.7 mm K^+^*versus* 20 mm K^+^; and δ*P* < 0.05 for 20 mm K^+^*versus* 20 mm K^+^ with aglycaemia.

With the exception of an increase in time constant and membrane resistance, aglycaemic conditions had no significant action on any of the passive or active membrane properties of the postganglionic ICG neurones; see Table 2. There was also no significant impact on synaptically mediated responses; see Fig. 7.

**Table 2:** Actions of high K^+^ and/or aglycaemia on the passive and active membrane properties of adult ICG neurones

	4.7 mm[K^+^]_o_	4.7 mm[K^+^]_o_, aglycaemia	20 mm[K^+^]_o_, aglycaemia
Passive properties			
*E*_m_ (mV)	−49.5 ± 7.6 (13)	−49.2 ± 7.0 (7)	−43.7 ± 5.5 (12)*‡
*R*_m_ (kΩ cm^2^)	7.8 ± 3.2 (12)	9.7 ± 3.8 (5)†	3.8 ± 1.7 (12)***‡
τ (ms)	7.8 ± 3.2 (12)	9.7 ± 3.8 (5)†	3.8 ± 1.7 (12)***‡
Active properties			
AP overshoot (mV)	18 ± 8.8 (10)	15 ± 11.7 (4)	11.3 ± 7.6 (9)**
AHP amplitude (mV)	16.9 ± 7.8 (10)	13.4 ± 9.7 (4)	6 ± 6 (9)***‡
AHP_50_ (ms)	23.9 ± 8.0 (10)	21.5 ± 5.8 (4)	33.9 ± 16.8 (8)^n.s.^

Values are means ±s.d.; number of neurones in parentheses. Repeated measures-ANOVA was used to test the significance between the groups.

* *P* < 0.05,

** *P* < 0.01 and

*** *P* < 0.005 for 4.7 mm[K^+^]_o_*versus* 20 mm[K^+^]_o_ with aglycaemia;

† *P* < 0.05 for 4.7 mm[K^+^]_o_*versus* 4.7 mm[K^+^]_o_ with aglycaemia;

‡ *P* < 0.05 for 4.7 mm[K^+^]_o_ with aglycaemia *versus* 20 mm[K^+^]_o_ with aglycaemia; and n.s., not significant.

The aglycaemic PSS solution was nominally glucose free. To measure the true glucose concentration postequilibration, we sampled PSS from ∼50 μm of the recorded neurone whilst superfusing normal, glucose-replete, PSS and aglycaemic, nominally glucose-free, PSS. The [glucose] in the superfusate in three instances in which this assay was performed was 10.0 mm (±0.6 mm) in normal PSS and 0.4 mm (±0.1 mm) in aglycaemic PSS.

### Combined high-K^+^ and glucose-free PSS

The passive membrane properties (*E*_m_, *R*_m_ and τ) of ICG neurones with the combined challenge of high-potassium (20 mm) and nominally glucose-free PSS were significantly different from the values recorded in high potassium alone. Considering active properties, only AHP amplitude was significantly different in these two conditions; see Table 3. Interestingly, superfusion of 20 mm K^+^ and nominally glucose-free PSS resulted in a decrease in frequency-dependent block of ganglionic transmission compared with that recorded in 20 mm K^+^ PSS see Fig. 7*E* and *F*. Thus, aglycaemia seems to mitigate the blocking action of high [K^+^] on high-frequency transmission (*P* < 0.05 at 10 and 20 Hz, 20 mm K^+^*versus* 20 mm K^+^ with aglycaemia, *n*= 11). In three of 11 neurones studied, ganglionic transmission was insensitive to the actions of high K^+^ and high K^+^ with aglycaemia. As a result, the defence action of high K^+^ with aglycaemia is restricted to 10–20 Hz.

## Discussion

The sympathetic and parasympathetic (vagal) divisions of the autonomic nervous system have antagonistic actions on cardiac function, and interaction between the two divisions is well established. Augmented, detrimental, sympathetic drive to the heart as part of the haemodynamic defence reaction to acute myocardial infarction has long been recognized (Katz, 2006). An indication that decreased synaptic transmission in intrinsic cardiac ganglia contributes to abnormal parasympathetic function in myocardial infarction comes from experimental models (Du *et al.* 1998; Kawada *et al.* 2002). Normally, the parasympathetic nervous system acts directly on the sinus node but in addition strongly inhibits sympathetic activity, activation of presynaptic muscarinic receptors on sympathetic terminals in the right atrium reducing the release of noradrenaline (Vizi *et al.* 1989)

Reduction of transmission in the ganglia will produce a sympathovagal imbalance. Thus, there will a predominance of sympathetic, pro-arrhythmic activity. A recent report has documented the role of the ICG in modulating extrinsic autonomic input to the heart and atrial fibrillation initiation (Hou *et al.* 2007).

During ischaemia, [K^+^]_o_ rises in the restricted spaces around ventricular cardiac muscle fibres (Carmeliet, 1999). The ICG neurones are commonly clustered in atrial fat-pads. To our knowledge, there are no comparable measurements of [K^+^]_o_ around ICG neurones during ischaemia. However, remote cardiac ischaemia can have an influence on populations of intrinsic cardiac neurons that are not in the ischaemic stress zone. For example, it has been demonstrated that neurones in the canine right atrial plexus receive mechano- and chemoreceptor information from sensory neurites in the ventricles (Thompson *et al.* 2000*a*). Altered activity in these neuronal processes will have consequences for the integrating processing activity of the ICG.

Hyperpolarizing current pulses evoked time-dependent rectification [the signature of *I*_h_ (H-current)] in all neurones in control (4.7 mm K^+^) PSS. Superfusion of kigh-K^+^ (20 mm) PSS abolished this behaviour. Previous studies on *I*_h_ in dissociated adult rat ICG neurones (using perforated-patch whole cell recordings) demonstrated that increasing [K^+^]_o_ from 3 to 15 mm increased the amplitude of *I*_h_ roughly threefold (Hogg *et al.* 2001). Current clamp recordings from dissociated adult rat ICG neurones demonstrated that increasing [K^+^]_o_ from 3 to 15 mm also inhibited the characteristic sag of the voltage response to hyperpolarizing currents, accompanied by a decrease in *R*_in_ (R.C. Hogg, A.A. Harper & D.J. Adams, unpublished data). A reduction in time-dependent rectification and an increase in *I*_h_ would seem counterintuitive. More requires to be found out about the other current(s) activated in high K^+^ to interpret this result.

The duration of after-hyperpolarization was altered in high-K^+^ PSS. This action was dependent on AHP_50_ duration (increased with AHP_50_ duration). Increased expression of the small calcium-activated K^+^ channel (SK_Ca_) currents underlying AHP is responsible for long AHP_50_ durations in these neurones (Rimmer & Harper, 2006). Confirmation of the identity of the mechanisms underpinning this difference in behaviour is clearly an important area for future investigation.

Ganglionic transmission in the set of neurones examined and the experimental conditions used in this study (physiologically relevant temperature and divalent ion levels) has a high safety factor, and is secure across the normal range of efferent discharge frequencies encountered (0.2–50 Hz; Kunze, 1972).

Intracardiac ganglia consist of several types of neurones, as well as small intensely fluorescent (SIF) cells. Three different neurone types within cardiac ganglia of adult animals have been identified according to their electrophysiological properties, the nature of the synaptic input they receive and morphological characteristics. Neurones which receive a local excitatory synaptic input and have been termed S (synaptic) or type I neurones (Selyanko, 1992; Edwards *et al.* 1995). The other two classes of cardiac neurones have been named type II neurones, which have been further divided into those which receive a direct, strong, excitatory efferent projection from the vagus and P (pacemaker like) neurones which receive no synaptic input but could be excited antidromically (Edwards *et al.* 1995). These two subtypes of type II neurones are multipolar and pseudounipolar or bipolar in morphology, respectively (akin to primary afferent neurones).

It must be emphasized that investigation of ganglionic transmission in the present study was limited to those neurones receiving a strong, suprathreshold input from the vagus, presumed to form efferent outflow to the atria. These neurones would be classified as principal cardiac neurones according to the scheme of Cheng & Powley (2000).

High extracellular potassium levels blunted ganglionic transmission at all frequencies. This could be the result of block of axonal conduction or suppression of exocytosis at the preganglionic nerve terminals. Block of antidromic (postganglionic) conduction is not totally convincing in discounting block of axonal conduction, since the properties of the postganglionic axon may not totally recapitulate those of the preganglionic axon. A good test to demonstrate this would be a progressive failure of synaptic transmission, with subthreshold EPSPs being recorded before block was complete. However, in practice when increasing K^+^, transmission quickly switched from secure to complete block. The reverse manoeuvre, changing from high-K^+^ to control PSS, did display progressive recovery, with subthreshold EPSPs being recorded before transmission became secure. This result suggests that the preganglionic action potential reaches the terminals and that transmitter release is suppressed.

The acute action of elevated potassium levels on peripheral components of autonomic control of the heart has been the subject of several studies. Modest elevations in [K^+^]_o_ decreased the negative chronotropic actions mediated by vagus nerve stimulation (Sears *et al.* 1999). This action was attributed to the influence of elevated extracellular K^+^ on the biophysical properties of the cardiac K_ACh_ channel. Similarly, high K^+^ attenuates the positive chronotropic action of sympathetic activation (Choate *et al.* 2001). It was argued that the high K^+^ accompanying challenges such as exercise or ischaemia may well have a protective action, abrogating the impact of sympathetic activation.

Aglycaemia (nominally glucose-free conditions) had no significant effect on the passive and active membrane properties of the postganglionic neurone. Similarly, aglycaemia did not affect ganglionic transmission.

Glucose was the metabolic substrate provided in the superfusing PSS; however, glucose is not the only energy substrate used by these tissues. Lactate is a major intermediate of glucose metabolism in normoxic neuronal tissue. It has been demonstrated that in the vagus nerve, both axons and Schwann cells take up glucose and lactate (Vega *et al.* 2003). According to this scheme, the glial cells take up more carbon fuel than they require and transfer the surplus to the neuronal elements. Satellite (glial) cells completely surround and are in close apposition to individual ICG neurones (Baluk & Gabella, 1987). Presumably, these satellite cells perform a similar metabolic function to that of other glial cells.

The combined challenge of high (20 mm) K^+^ and aglycaemia was less than the action of 20 mm K^+^ for many of the parameters measured, both passive membrane properties and ganglionic transmission. It would seem that algycaemia may blunt or provide a defense against the ganglionic blocking actions of high extracellular potassium. Clearly, with presently available information the reasons underpinning this shielding activity must remain speculative.

### Physiological significance

These data indicate that the presynaptic terminal is the primary target for high K^+^ and aglycaemia in the ICG. While a moderate increase of [K^+^]_o_ (∼10 mm) changed the passive and active properties of the ICG neurones, it did not influence efferent vagal ganglionic transmission. Similar elevations of K^+^ have been recorded in arterial plasma (∼8 mm) during strenuous exercise (Medbo & Sejersted, 1990). Intense changes (20 mm), akin to those encountered during ischaemia, blocked efferent synaptic transmission in the ICG. Identification of the channels or membrane transport systems underpinning the sensitivity of the presynaptic membrane to these components of ischaemia may lead to the development of new therapeutic tools to restore parasympathetic control during myocardial ischaemia.
